# Health inequalities at the intersection of multiple social determinants among under five children residing Nairobi urban slums: An application of multilevel analysis of individual heterogeneity and discriminatory accuracy (MAIHDA)

**DOI:** 10.1371/journal.pgph.0002931

**Published:** 2024-02-29

**Authors:** Eliud Kibuchi, Ivy Chumo, Caroline Kabaria, Helen Elsey, Penelope Phillips-Howard, Noemia Teixeira de Siqueira-Filha, Lana Whittaker, Alastair H. Leyland, Blessing Mberu, Linsay Gray

**Affiliations:** 1 School of Health and Wellbeing, MRC/CSO Social and Public Health Sciences Unit, University of Glasgow, Glasgow, United Kingdom; 2 African Population and Health Research Center, Nairobi, Kenya; 3 Department of Health Sciences, University of York, York, United Kingdom; 4 Liverpool School of Tropical Medicine, Pembroke Place, Liverpool, United Kingdom; PLOS: Public Library of Science, UNITED STATES

## Abstract

In this analysis we examine through an intersectionality lens how key social determinants of health (SDOH) are associated with health conditions among under-five children (<5y) residing in Nairobi slums, Kenya. We used cross-sectional data collected from Nairobi slums between June and November 2012 to explore how multiple interactions of SDoH shape health inequalities in slums. We applied multilevel analysis of individual heterogeneity and discriminatory accuracy (MAIHDA) approach. We constructed intersectional strata for each health condition from combinations of significant SDoH obtained using univariate analyses. We then estimated the intersectional effects of health condition in a series of MAIHDA logistic regression models distinguishing between additive and interaction effects. We quantified discriminatory accuracy (DA) of the intersectional strata by means of the variance partitioning coefficient (VPC) and the area under the receiver operating characteristic curve (AUC-ROC). The total participants were 2,199 <5y, with 120 records (5.5%) dropped because health conditions were recorded as “not applicable”. The main outcome variables were three health conditions: 1) whether a child had diarrhea or not, 2) whether a child had fever or not, and 3) whether a child had cough or not in the previous two weeks. We found non-significant intersectional effects for each health condition. The head of household ethnic group was significantly associated with each health condition. We found good DA for diarrhea (VPC = 9.0%, AUC-ROC = 76.6%) an indication of large intersectional effects. However, fever (VPC = 1.9%, AUC-ROC = 66.3%) and cough (VPC = 0.5%, AUC-ROC = 61.8%) had weak DA indicating existence of small intersectional effects. Our study shows pathways for SDoH that affect diarrhea, cough, and fever for <5y living in slums are multiplicative and shared. The findings show that <5y from Luo and Luhya ethnic groups, recent migrants (less than 2 years), and households experiencing CHE are more likely to face worse health outcomes. We recommend relevant stakeholders to develop strategies aimed at identifying these groups for targeted proportionate universalism based on the level of their need.

## Introduction

Rapid urbanisation and population growth, which is largely unplanned and unregulated, has led to growing numbers of people living in slums in low- and middle-income countries (LMICs). Slum areas are characterised by inadequate access to safe water, inadequate access to sanitation and other infrastructure, poor structural quality of housing, overcrowding, and insecure residential status, which are risk factors for illnesses [1–4]. Currently, over one billion people live in slums with more than 80% attributed to regions in Asia and Sub-Saharan Africa [5]. In the Kenyan context, 2.5 million people live in 200 slum settlements in Nairobi representing 56% of the city’s population, yet slums only occupy 6% of the residential area [6, 7]. Children in slums tend to have worse health conditions (i.e., health inequalities) compared to the rest of the city and rural areas [3, 4, 8–10]. For example, child mortality in Nairobi slums is 1.3 times higher when compared to other areas of city due to health conditions such as pneumonia, malaria, HIV/AIDS, diarrhea, and malnutrition [8, 10, 11]. Efforts are necessary to create an evidence-base for informing policies for effective urban health programming by state and non-state actors to reduce children under-five (<5y) health inequalities in slums [12]. Strikingly, this will challenge the notion of “urban advantage” where <5y living in slums are often deemed to benefit from the abundant resources and strong infrastructure found in cities [13]. This will allow for priority setting in tackling health inequalities experienced these settings.

The most common causes of mortality and morbidity for <5y in Nairobi slums are diarrhea, malaria, and pneumonia/acute respiratory distress/cough [2–4, 14–16]. For example, <5yr mortality rate in Nairobi slums is 3.6 times higher than that of the Nairobi as a whole [17]. In the same study, it was found that prevalence of common children’s illnesses in slums was higher compared to both rural and urban areas [17]. The percentage prevalence for diarrhea, cough and fever in Nairobi slums were 20.2, 24.6 and 17.2 compared to 15.6, 5.9 and 18.7 in the whole of Nairobi respectively [17].The health of <5y not only depends on broad living conditions of slums but also on health and welling of their mothers and their families [2–4, 18]. For example, fertility rate in Nairobi slums is 3.5 which is higher than 2.7 of the whole Nairobi and this tends to have a direct adverse effect in terms of provision of better care [10, 17]. One of the causes of higher fertility rate in Nairobi slums is the lower median age of 20.0 years at first birth compared to 22.2 years of Nairobi as whole [17].

Moreover, differences in social and economic positions and power among different ethnic groups residing in Nairobi slums may contribute to distinct patterns of exposure and health problems [19, 20]. For example, children aged 12–23 months born of mothers of Luhya tribe were found to have the highest prevalence of fever at 21.6% compared to Kamba at 14.1%. These differences are mostly attributed to rural-urban linkages and land ownership whereby slum communities near their rural homes can supplement their income and livelihoods [21–23]. For example, Kamba and Kikuyu ethnic groups whose regions of origin are close to Nairobi are most likely to maintain contacts with their rural homes compared to Luo and Luhya [21]. This close contact with their rural areas provides an avenue to rely on both nuclear and extended families for financial and social support which improves the overall health status of their children through improved nutrition and ability to access healthcare [24–27]. However, over the last decade mobile money transfer services have increased financial activity between rural and urban areas which have contributed to addressing poverty and inequality among slum dwellers [28].

Therefore, to address the health conditions for <5y in slums requires a clear understanding of how social determinants of health (SDOH) intersect to create complex and unique social positions of vulnerability. That is, different SDoH factors come together in a complex and interactive way which leads to an increase in the risk of adverse health conditions in certain social groups or positions [29]. SDoH are described as the conditions in which people live, grow, learn, work, play, and age [30, 31]. This has led to the growing recognition in the past decades of the roles of SDoH in shaping not only the individuals, but also the population’s heath [32]. Accordingly, social contextual factors such as gender discrimination can produce disparities in terms of education, employment, income, and wealth, which leads to social stratification visibly expressed as differences in access to material resources like health, transportation, or housing, which in turn leads to further worsening of social context, and the cycle perpetuates [31].

The literature on determinants that influence <5y health conditions for slum dwellers is mixed [33–35]. Some of the factors that have been identified to be associated with poor health include child sex [33, 34, 36], child age [36–38], maternal education [35–37], income and wealth [36], tenure [33] among others. However, due to interlocking systems of privilege and disadvantage of these determinants in terms of health conditions, the experience of <5y living in a particular intersection cannot be understood by looking at each factor independently [39, 40]. Therefore, it becomes important to provide a deeper insight about the influence of multidimensional and multiplicative impact of these determinants categories on <5 y health inequalities [35, 40, 41]. This can be achieved by utilising intersectionality theory which posits that social identities and positions are interdependent and mutually constitutive [42–44]. Such proper understanding of the SDoH that influence <5y health in slums can help transform power relations through development of child-responsive interventions tailored to local community’s needs. However, this is is hindered by the huge gap in the availability of local quality data from slum settings [45] and limited uptake of quantitative intersectionality in social epidemiology [35, 41, 44, 46, 47].

To fill this knowledge gap and inform such action, we aim to systematically examine how various SDoH factors intersect to either worsen or improve the health of <5y living in Nairobi slums through intersectionality lens. Intersectionality lenses provide a systematic approach to understand drivers of health conditions for <5y based on their unique positioning within a web of interacting SDoH [48–50]. Intersectionality allows to account for the complexity of the real world in understanding how different SDoH influence health inequities through marginalisation and privilege in a multiplicative and interactive ways [50–52]. The choice of intersectionality theory is informed by the Accountability and Responsiveness in Informal Settlements for Equity (ARISE) consortium which uses this framing to analyse and address the complex social, economic, and political systems that shape health and wellbeing outcomes amongst marginalised urban populations [53].

In this study, we aim to investigate how SDoH affect three health conditions (i.e., diarrhea, fever and cough) for <5y in Nairobi slums within an intersectionality framework using multilevel analysis of individual heterogeneity and discriminatory accuracy (MAIHDA) [52, 54]. MAIHDA approach ascertains intersectional associations by identifying social groups from multiple interactions that are disproportionately discriminated against and disadvantaged than would be expected in absence of these intersections [52, 54, 55]. Utilisation of MAIHDA will help explore associations of intersectional social positions and identities by considering them as contextual factors of interlocking socio determinants of health. This aligns well with the current understanding of how SDoH influence health inequalities since it involves transforming individual, household, and community characteristics into contextual social strata that represent an interlocking system of oppression and vulnerability leading to inequalities [42, 56].

To date, fixed effects models with interaction terms have been used to explore additive and multiplicative associations of SDoH and health outcomes [54]. However, fixed effect models are often limited to fully exploring the many dimensions of social identities due to sample size and model parsimony [52, 54, 57]. Moreover, the use of reference groups limits the ability to explore crucial patterns of inequalities among those groups who experience combination of privilege and disadvantage and reinforce the notion of “default” groups contributing to further marginalisation [54]. MAIHDA approach addresses all these limitations and helps explore intersecting dimensions of social identity and positions and how they create inequalities and inequities through patterning [52, 54, 58, 59]. In turn, this helps identify the most likely vulnerable groups in society for policy intervention.

Therefore, this study used MAIHDA modelling approach to explore intersectional inequalities in health outcomes (i.e., diarrhea, fever, and cough) among <5y living in Nairobi slums. This will inform how social identity and position shape health inequalities and inequities among this group and determine the extent at which these social identities and positions explain health inequalities for effective policy and programme development. We used data from Nairobi Cross-section Slums Survey (NCSS) 2012 [10].

## Methods

### Study design and sampling

Data for this paper were generated from the Nairobi Cross-section Slums Survey (NCSS) 2012, collected by the African Population and Health Research Center (APHRC) from all slums in Nairobi between June and November 2012 using face-to-face interviews [10]. The NCSS 2012 is one of the few slum surveys in the global south and contains SDoH factors which will help analyse complex social and economic systems that shape health conditions among marginalised urban populations which is not possible with most routine health data in slums. The sample included in the survey was calculated based on the proportion of children 12–23 months who had been fully immunised in the poorest wealth quintile (i.e., 65.9%) using a margin of error of 0.03, design effect of 1.50 and critical value of α = 0.05 [10]. The number of households required to estimate the percentage of children 12–23 months fully vaccinated was large enough to allow estimation of the other indicators such as diarrhea, fever, and cough with the specified precision [10]. Questions on child health in NCSS 2012 were contained in women’s questionnaire on the module “child immunization and child health” which was administered to women aged 12 to 49 years in the sampled households. Child’s mother was asked whether the child had an occurrence of either diarrhea, cough and fever within the period of two weeks preceding the survey.

A two-stage random sampling methodology was used to select the households to be interviewed. The first stage involved selecting 30% of the sampled enumeration areas using the probability proportional to population size (PPP) sampling. This was followed by drawing a random sample consisting of 35% of households listed in each selected EA which resulted to 6,450 households of which 314 vacant structures were dropped. The final sample of interviewed households was 6,268 households with 5,490 households successfully interviewed yielding a response rate of 88% [10]. A total of with 4,912 women aged 12–49 years were eligible to be interviewed and 4,240 were successfully interviewed yielding a response rate of 86%. Women with least one child aged <5yr were 1, 509 (35.6%) of the number interviewed, with only 46 (1.1%) from the same households and therefore not expected to influence the overall estimates. Written and informed consent was obtained for all interviews with additional consent obtained for participants aged less than 18 years from either parents or guardians. In total, 2,199 records for health data on <5y were provided in the child immunization and child health module in the women’s questionnaire. However, 120 records (5.5%) were dropped because child health conditions were recorded as “not applicable” leaving an analytical sample of 2,079.

### Ethical approval

The study used data from the Nairobi Cross-sectional Survey 2012 (NCSS2012) doi:11239/176-2014-026-1.0 which excluded any participant identifiers. Ethical approval for the NCSS 2012 study was obtained from the Kenya Medical Research Institute’s Ethics Review Committee.

### Patient and public involvement

Members of the public were not involved in the study concept or design since we used secondary data.

### Measures

The dichotomous outcome variables were three health conditions or symptoms which mostly affect <5y in slums: 1) whether a child had diarrhea or not, 2) whether a child had fever or not, and 3) whether a child had cough or not. The explanatory variables examined in the study were based on factors cited in the literature as influencing health conditions for <5y [2, 60–62]. In addition, discussions were held with the APHRC -ARISE team who are well grounded in the context of where the data were collected. The analytical approach used in this paper requires all explanatory variables to be categorical which necessitated the need to collapse continuous variables into categories. The explanatory variables were classified into four groups:

1. Children’s demographics: age (*1 year and less*, *2–5 years)*; and sex (*female or male)*.
2. Head of household characteristics: sex (*male or female*), ethnic group (*Kamba*, *Kikuyu Luhya*, *Luo*, *and others)*; age (*17–24 years*, *25–34 years and 35 and over years*) and education status (*none*, *educated and don’t know/not applicable)*.
3. Mother’s characteristics: age (*18 years and below*, *19–49 years); and education (primary*, *post primary*, *none)*.
4. Social structure characteristics of the household: wealth index (*rich*, *middle and poor*), length of stay (*new migrants (i*.*e*.*2 years and less)*, *old migrants (i*.*e*. *more than 2 years)*, *not applicable/missing*); religion (*catholic*, *protestant and others*); tenure (*no rent paid or pays rent*), food security (*enough or not enough*); health insurance (*no or yes*); income generating activity (*employed*, *missing/not applicable and own business*); and catastrophic health expenditures (CHE) at 40% threshold (*no or yes*).

The categorisation of mother’s age aimed to differentiate between child and adult mothers since the age of sexual consent in Kenya is 18 years and any births that occur before this age are considered as from child mothers.

The Principal Components Analysis (PCA) was used to generate a wealth index based on the asset approach for measuring socio-economic position using source of drinking water, type of toilet facility, cooking fuel used, lighting type at night, material used to construct floor, wall and roof of dwelling, and household possessions (ownership of household items) variables [10]. In these analyses we collapsed wealth index from five categories into three for easier interpretation. Catastrophic health expenditure (CHE) was computed based on the approach detailed by [63, 64] using the empirical methodological procedure used by [65]. We considered that a household experienced CHE if their total payment equalled or exceeded 40% of the household’s capacity to pay on non-subsistence spending [64]. The variables used to compute CHE were household’s total consumption expenditure, food expenditure, out of pocket health expenditure, and the size of household. We dealt with missing data by undertaking complete case analyses.

### Statistical methods

We applied MAIHDA to estimate intersectional inequalities separately in the children’s health conditions (i.e., diarrhea, fever, and cough) [52, 54, 55, 57, 66, 67]. Intersectional strata were defined by combinations of variables (e.g., sex, age, wealth index, education etc.) which allowed decomposition of the differences in children health conditions into within and between strata [68]. However, there is no established framework that lists determinants associated with <5y health conditions in slums based on their importance. Therefore, we adopted a model building process to select factors used in creating social strata based on their significance. The choice of variables used to construct intersectional strata for each health condition was based on results from univariate analysis of each variable [69]. Only variables that were significant (p<0.05) in the univariate analysis were used to construct intersectional strata and their correlations assessed using Cramér’s V to avoid multicollinearity [70]. It is important to note that inclusion of only statistical significance variables in the creation of social strata does not necessary imply that other variables lacked practical importance on their association with health conditions. This parsimonious approach was informed by the need to overcome the issue of the large number of strata in the case of all available variables being included leading to unstable estimates. Inevitably, some variable combinations had zero individuals resulting in a reduced number of strata. This represented a clear multilevel structure with <5y (level 1) nested within intersectional strata (level 2) which can be viewed as analogous to a type of context such as neighborhood.

Using the MAIHDA framework, we assessed discriminatory accuracy (DA) and intersectional estimates through two successive multilevel logistic regression models. Model 1 investigated whether there was significant clustering within intersectional strata. This model did not include any explanatory variables and only contained an intercept to estimate the mean health condition and a random effect used to model intersectional strata differences (i.e., variance). Model 2 explored to which extent intersectional strata differences were explained by SDoH used to construct intersectional strata. Model 2 was an extension of model 1 and involved adjusting for variables used to construct intersectional strata as main effects. Main effects were used to estimate model regression coefficients which were presented as odds ratio and described the association between the SDoH variables and <5y health conditions. In the absence of intersectional strata differences, main effects used to construct strata are expected to completely explain intersectional strata differences obtained in model 1 implying that strata random effects in model 2 are equal to zero. However, if strata random effects in model 2 are not equal to zero and assuming no relevant variables were omitted on the model it indicates existence of intersectional associations between variables. In addition, we fitted model 3 to account for possible variation in health outcomes by adjusting for all explanatory variables considered in univariate analysis as main effects.

The measure used to estimate DA of intersectional strata was the variance partitioning coefficient (VPC) [68, 71]. VPC (also known as intraclass correlation) quantifies the share of the total individual variance in having a health condition that is accounted for at the intersectional strata level [71]. We presented VPC as the percentage share of the individual variance which lies within intersectional strata by multiplying it by 100. The VPC values higher than 5% indicate acceptable DA [72]. In addition, we assessed DA using area under the receiver operating characteristic curve (AUC -ROC) which measures the ability of model to classify <5y with or without health condition as a function of their predicted probabilities [55, 73]. The AUC-ROC represents an overall accuracy of model and is bounded between 0.5 and 1. A value of 0.5 indicates that model predictions are no better than random guessing meaning that explanatory variables used in the model have no predictive power, while a value of 1 represents perfect discrimination between <5y with or without health condition. The proportional change in variances (PCV) between models 1 and 2 and models 1 and 3 were computed to quantify the proportional of the total between-stratum intersectional variance detected in model 1 that was explained after adding fixed effects in models 2 and 3 [55, 72, 73]. The lower the PCV, the higher the amount of unexplained variance which can be due to either interaction effects or omitted variables in the model. Detailed description of model 1, model 2, VPC and PCV are provided in S1 Text.

Intersectional estimates were presented for both model 1 and model 2 with a positive estimate implying that <5y in that intersectional stratum have a higher risk than expected based on the addition of the risks associated with the variables that define the intersectional stratum. On the other hand, a negative intersectional estimate means a lower risk than expected. Intersectional estimates and their corresponding 95% credible intervals (CIs) ranked from lowest to highest were presented as plots. It is important to note that MAIHDA approach is not limited by sample size of intersectional strata which enables exploration of intersectionality using many dimensions of social positions and identities.

We applied Stan for full Bayesian inference through Markov Chain Monte Carlo (MCMC) methods to fit MAIHDA models [74]. The models were fitted using brms package for Bayesian multilevel models using Stan (version 2.16.1) [75] in R (version 4.1.1); with R codes available on https://github.com/Kibuchi-eliud/Health_inequalities_for_under_five_in_slums_intersectionality.git in GitHub [76]. We used weakly informative priors and ran all analyses using 20,000 iterations with a burn-in period of 2,000. MCMC chains were checked graphically for convergence.

## Results

### Diarrhea

The final complete case analysis sample for diarrhea had 1,738 records after 341 (16.4%) records were dropped; of which 170 (8.2%) were due to diarrhea outcomes missing and 171 (8.2%) had missing values in variables considered for intersectional strata. The number of <5y with diarrhea were 304 (17.5%) and those without 1,434 (82.5%); the distribution of SDoH by diarrhea is provided in S1 Table. Results for univariate analyses which informed the selection of variables to be included in creating intersectional strata for diarrhea are also in S2 Table. From S2 Table, the variables which were significant and subsequently used to create intersectional strata and included in model 2 were: child age, head of household ethnic group and education, wealth index, length of stay, health insurance, religion, food security, and tenure. The intersectional strata created for diarrhea were 751 with only 80 (10.6%) strata having 5 or more <5y per stratum.

Table 1 presents model 1, 2 and 3 results for all health outcomes. For diarrhea, Table 1 results for model 2 indicate elevated significant odds of getting diarrhea for children aged 2 to 5 years compared to those aged 1 year and younger after controlling for other factors. The odds of getting diarrhea in <5y from educated households and those who provided no answer were elevated and significant compared to those from households with no education. The odds of getting diarrhea for <5y coming from middle wealth groups were 1.7 times higher and significant compared to those in the richest category when controlling for other factors. For ethnic group, the odds of <5y from Luhya and Luo headed households getting diarrhea were significant and 1.9 and 1.6 times higher respectively compared to <5y from Kamba community when controlling for other factors. Children from old migrants’ households have lower and significant odds of getting diarrhea compared to those from new migrants. Finally, <5y from households with health insurance had lower and significant odds of getting diarrhea compared to those from households with no insurance when controlling for other factors. The odds of getting diarrhea obtained in model 2 were similar to those obtained in model 3 after controlling for all variables.

**Table 1:** Estimates from multilevel models of diarrhea, cough and fever among under-five children living in Nairobi informal settlements with observations clustered by social strata.

		Diarrhea			Fever			Cough		
		Model 1	Model 2	Model 3	Model 1	Model 2	Model 3	Model 1	Model 2	Model 3
	Category (ref)	OR (95%CI)	OR (95%CI)	OR (95%CI)	OR (95%CI)	OR (95%CI)	OR (95%CI)	OR (95%CI)	OR (95%CI)	OR (95%CI)
Intercept		0.18(0.15, 0.21)	0.03(0.00,0.12)	0.03(0.01, 0.14)	0.20(0.17,0.23)	0.13(0.05, 0.3)	0.21(0.05,0.78)	0.34(0.29,0.40)	0.20(0.12,0.34)	0.11(0.03,0.38)
Child age	1 year and less (ref)		1.00	1.00			1.00			1.00
	2–5 years		0.69(0.51,0.93)	0.70(0.51, 0.95)			0.86(0.65,1.14)			0.92(0.72,1.18)
Child Sex	Female (ref)			1.00			1.00			1.00
	Male			1.33(1.01, 1.75) *		0.79(0.59, 1.05)	0.76(0.56,1.01)			0.90(0.71,1.13)
Women age	18 years and less (ref)			1.00		1.00	1.00			1.00
	19–49 years			0.83(0.48, 1.46)		0.71(0.44, 1.16)	0.81(0.49,1.37)			1.06(0.66,1.73)
Women education	Primary (ref)			1.00			1.00			1.00
	Post primary			0.97(0.73, 1.30)			1.00(0.76,1.33)			0.96(0.75,1.23)
	None			1.05(0.26, 3.47)			0.00(0.00,0.00)			0.26(0.05,0.89)
Head of household sex	Female (ref)			1.00			1.00			1.00
	Male			0.80(0.52, 1.23)			1.22(0.79,1.92)			1.10(0.76,1.62)
Head of household age	17–24 (ref)			1.00			1.00			1.00
	25–34			1.33(0.79, 2.28)			0.89(0.54,1.48)			1.02(0.66,1.60)
	35 and above			1.20(0.69, 2.13)			0.70(0.41,1.22)			0.80(0.50,1.30)
Head of household Ethnic	Kamba (ref)		1.00	1.00		1.00	1.00		1.00	1.00
	Kikuyu		1.20(0.70,2.02)	1.22(0.71, 2.10)		1.53(0.89, 2.65)	1.47(0.83,2.65)		1.61(1.03,2.55)	1.71(1.06,2.78)
	Luhya		1.85(1.16,3.00) *	1.88(1.17, 3.07) *		2.91(1.82, 4.80) *	3.15(1.92,5.34) *		2.63(1.75,4.08) *	2.75(1.78,4.44) *
	Luo		1.62(1.00,2.66) *	1.65(1.00, 2.74) *		2.04(1.23, 3.43) *	2.05(1.21,3.50) *		2.27(1.49,3.55) *	2.32(1.49,3.70) *
	Other		1.24(0.72,2.12)	1.28(0.74, 2.22)		2.44(1.45, 4.19) *	2.69(1.56,4.72) *		2.27(1.45,3.57) *	2.45(1.52,3.99) *
Head of household education	None (ref)		1.00	1.00			1.00			1.00
	Educated		2.25(1.19,4.57) *	2.38(1.19, 4.98) *			0.83(0.48,1.47)			1.23(0.73,2.10)
	Don’t know/not applicable		2.46(1.27,5.15) *	2.70(1.36, 5.74) *			0.78(0.44,1.38)			1.19(0.71,2.02)
Wealth index	Rich (ref)		1.00	1.00		1.00	1.00		1.00	1.00
	Middle		1.71(1.19,2.47) *	1.70(1.17, 2.47) *		1.38(0.97, 1.95)	1.36(0.93,1.96)		1.29(0.94,1.75)	1.26(0.89,1.76)
	Poor		1.37(0.96,1.97)	1.32(0.91, 1.93)		1.17(0.83, 1.63)	1.21(0.84,1.73)		1.19(0.88,1.59)	1.23(0.87,1.69)
Length of stay	New migrants (ref)		1.00	1.00		1.00	1.00		1.00	1.00
	Old migrants		0.73(0.44,1.22)	0.71(0.42, 1.20) *		0.77(0.48, 1.23)	0.82(0.49,1.35)		0.63(0.41,0.95) *	0.62(0.40,0.97) *
	Missing		0.64(0.40,1.05)	0.63(0.38, 1.05)		0.66(0.42, 1.05)	0.70(0.43,1.17)		0.67(0.45,1.01)	0.74(0.48,1.15)
Religion	Catholic (ref)		1.00	1.00			1.00			1.00
	Protestant		1.34(0.95,1.92)	1.38(0.97, 1.98)			0.84(0.61,1.16)			1.00(0.76,1.33)
	Other		0.79(0.42,1.42)	0.88(0.43, 1.73)			0.78(0.41,1.45)			0.85(0.49,1.47)
Health insurance	No (ref)		1.00	1.00			1.00			1.00
	Yes		0.70(0.49,0.98) *	0.69(0.48, 0.99) *			0.94(0.68,1.30)			1.11(0.85,1.46)
Disability	No (ref)			1.00			1.00			1.00
	Yes			1.55(0.50, 4.31)			1.03(0.32,2.90)			1.35(0.52,3.29)
	Missing/not applicable (ref)			0.80(0.39, 1.59)			1.10(0.58,2.04)			1.07(0.62,1.83)
Tenure	No rent paid (ref)			1.00			1.00			1.00
	Pays rent		1.82(0.86,4.15)	1.99(0.92, 4.62)			0.65(0.36,1.21)			1.26(0.73,2.23)
Food security	Enough (ref)		1.00	1.00		1.00	1.00			1.00
	Not enough		1.26(0.87,1.84)	1.25(0.86, 1.85)		1.28(0.89, 1.87)	1.36(0.93,2.01)			1.02(0.76,1.38)
Income generating activity	Employed (ref)						1.00			1.00
	Own business			0.85(0.58, 1.22)			1.12(0.65,1.90)			1.11(0.70,1.73)
	Missing/Not applicable			0.72(0.40, 1.25)			1.20(0.84,1.73)			1.00(0.73,1.37)
Catastrophic health expenditure	No (ref)			1.00		1.00	1.00		1.00	1.00
	Yes			1.16(0.70, 1.89)		1.99(1.28, 3.07)	1.91(1.20,2.99)		1.94(1.31,2.88) *	2.05(1.36,3.09) *
Strata variance		0.41	0.32	0.36	0.24	0.06	0.08	0.11	0.09	0.03
Strata N		751	751	751	282	282	282	78	78	78
Individual		1,738	1,738	1,738	1,731	1,731	1,731	1,721	1,721	1,721
AUC -ROC		88.40%	76.57%	77.00%	76.00%	66.17%	66.17%	63.80%	61.65%	61.65%
VPC		11.00%	9.00%	9.86%	6.54%	1.86%	2.66%	3.16%	0.51%	0.87%
PCV			19.93%	11.00%		72.87%	60.94%		84.39%	73.30%

Model 1: empty model; model 2: main effects model; model 3: model adjusted for all explanatory variables; OR (95%CI): Odds Ratio (95% Confidence Interval);

*: significant at 5% level of significance; AUC-ROC: Area under the receiver operating characteristic curve; VPC: variance partitioning coefficient; PCV: proportional change in variance.

*VPC* = [*variance between social strata/(variance between social strata* + 3.29)] × 100

*PCV* = [(*variance between social strata in model* 1 − *variance between social strata in model* 2)/*variance between social strata in model* 1] × 100.

**Fig 1:**
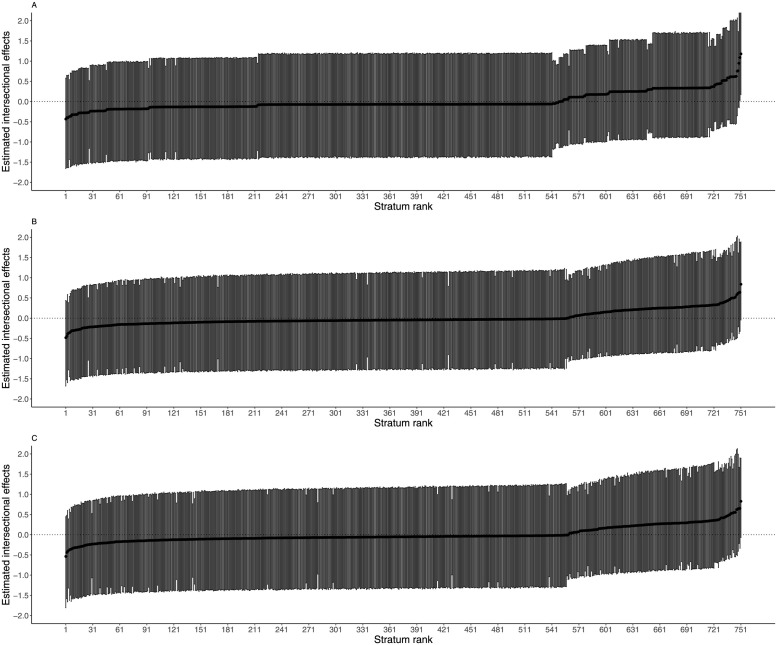
Estimated intersectional effects estimates and their corresponding 95% credible intervals (CI) for each stratum for diarrhea ranked from lowest to highest: Model 1 (panel A), model 2 (panel B), and model 3 (panel C).

The AUC-ROC for model 1 was 88.4%, suggesting excellent discriminatory accuracy. The VPC was 11.9% indicating that approximately 12% of the total variance is explained by the clustering of social strata. an. The AUC-ROC dropped to 76.6% and 77.0% and VPC to 9.0% and 9.9% after controlling for main effects in models 2 and 3 respectively indicating an existence of intersectional multiplicative effects in relation to variables included. The PCV was 19.9% and 11.0% for models 2 and 3, indicating that 80.1% and 89.0% of variance was not explained by adding main effects respectively which can be attributed to intersectional effects assuming no important variables were omitted in model 2. This indicates that differences between strata were due to intersectional effects of the variables used to create intersectional strata indicating the SHoD influence the probability of >5yr getting a diarrhea or not. Sensitivity analysis results where we excluded individuals with “Don’t know and not applicable” in education variable (S3 Table and S1 Fig) were similar those obtained in Table 1 and Fig 1 suggesting a decreased level of potential bias for missing data on overall pattern.

Fig 1 presents intersectional estimates for diarrhea with their corresponding 95% CI ranked from lowest to highest. The top Panel A shows that estimates for model 1 ranged from –0.4 to 1.2 with only 22 (2.9%) of 751 intersectional strata’s 95% CI not crossing zero (on far left), indicating significant intersectional estimates. Panel B and C represent intersectional estimates for model 2 and 3 which ranged from -0.5 to 0.8 and all their 95% CI crossed zero. This indicates that although strata associations exist for diarrhea among the children even after accounting for fixed effects used to create these intersectional strata, the intersectional estimates obtained are not significantly different across different strata. However, strata with children aged 1 year and younger from Luhya and Luo ethnicities, new immigrants, with no health insurance and from educated households are at a higher risk of diarrhea compared to other intersectional strata.

### Fever

For fever, the final analysis sample had 1,731 records after 348 (16.7%) records were dropped; of which 189 (9.1%) were due to fever outcomes missing and 159 (7.6%) had missing values in variables used to construct intersectional strata. The number of <5y with fever were 296 (17.1%) and those without 1,435 (82.9%), with the distribution of SDoH by fever provided in S4 Table. Intersectional strata were constructed using child gender, head of household ethnic group, woman’s age, wealth index, CHE, length of stay and food security variables which were significant in univariate analyses (S5 Table). Overall, models 2 and 3 in Table 1 shows that odds of fever among <5y from Luhya, Luo and other ethnicities were significantly higher compared to the Kamba ethnic group. The odds of getting fever for <5y from households which experience CHE were 2 times higher compared to those from households with no CHE.

The AUC-ROC for model 1 was 75.9% indicating an acceptable DA. The VPC for model 1 was 6.5% which dropped to 1.9% and 2.7% in models 2 and 3after adding main effects which indicates low DA. The AUC-ROC dropped to 66.2% for both models 2 and 3 indicating poor DA which may be caused by the poor definition of social strata based on selected variables which do not adequately account for fever inequalities. The PCV for fever was 72.5% and 60.9% for model 2 and 3 respectively indicating that 27.5% and 39.1% of variance was not explained by adding main effects which suggests that some differences between strata can be attributed be to intersectional strata.

**Fig 2:**
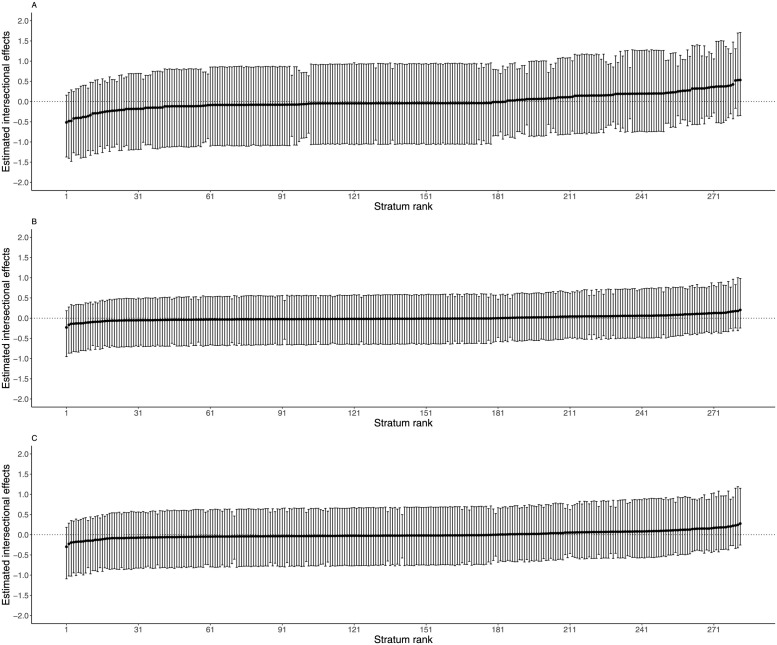
Estimated intersectional effects estimates and their corresponding 95% credible intervals (CI) for each stratum for fever: Model 1(panel A),model 2 (panel B), and model 3 (panel C).

Fig 2 (panel A) indicates that intersectional effects ranged from -0.51 to 0.53. In Panel B and C for models 2 and 3, intersectional estimates ranged from -0.23 to 0.21 and -0.30 to 0.30 respectively. In models 1,2 and 3, all 95% CI estimates crossed zero indicating no significant intersectional effects in any intersectional stratum. However, intersectional strata with <5y from Luhya, Luo, and other ethnic groups and from households experiencing CHE are at a higher risk of fever compared to other strata.

### Cough

The sample for cough had 1,721 records after 358 (17.2%) records were deleted; of which 204 (9.8%) were due to cough outcomes missing and 154 (7.4%) had missing values in variables used to construct intersectional strata. The number of <5y with cough were 430 (25.0%) and those without 1,291 (75.0%) with the distribution of SDoH by cough provided in S6 Table. The number of intersectional strata in cough models were 78. They were constructed using the variables: head of household ethnic group, wealth index, length of stay and CHE chosen based on univariate analyses (S7 Table).

Table 1, models 2 and 3 show that there was strong evidence for an association of head of household ethnic group and household’s CHE with a child’s cough. Compared to the Kamba ethnic group, the odds of getting cough were significantly higher (more than 2 times higher) in the Luhya, Luo, and other ethnic groups. Children from old migrants’ household have lower and significant odds of getting cough compared to those from new migrants. The odds of having a cough were 1.9 times higher for <5y from households experiencing CHE compared to the respective reference group. In addition, the odds of getting cough were lower in old migrants compared to new migrants.

The VPC in model 1 indicates 3.2% of the total variance among under five with and without cough was located at the intersectional strata level an indication of low DA. The AUC-ROC for model 1 was 63.8%, suggesting poor DA. The VPC and AUC-ROC dropped to 0.5% and 61.8% respectively in model 2 after adding main effects which indicates a low DA probably due to poor definition of social strata. PCV for cough in model 2 was 84.9% indicating that only 15.1% of intersectional variance was not explained by adding main effects. This suggests that only small differences between strata were due to interaction of the variables used to create intersectional strata. The results for cough in models 2 and 3 were similar.

**Fig 3:**
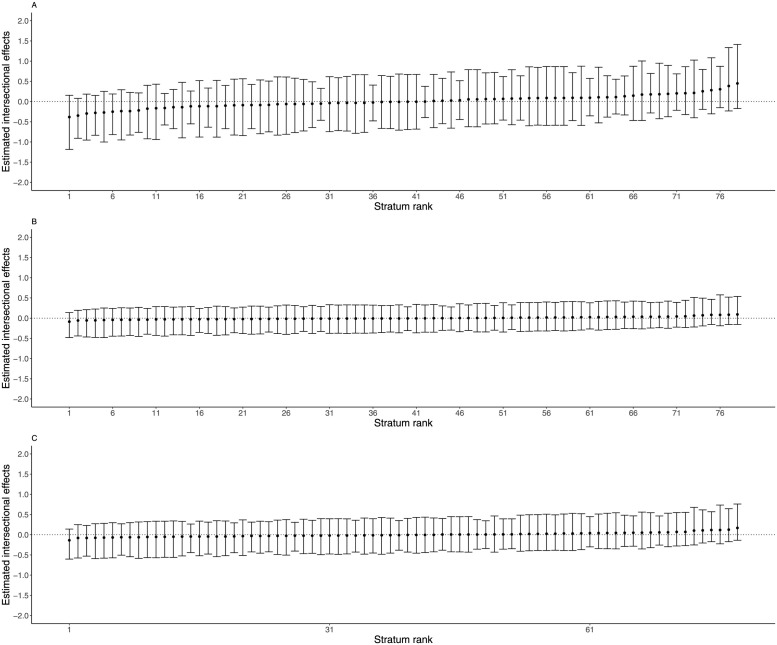
Estimated intersectional effects estimates and their corresponding 95% credible intervals (CI) for each stratum for cough: Model 1(panel A), model 2 (panel B), and model 3 (panel C).

Fig 3 indicates that intersectional effects for model 1 (panel A) ranged from -0.38 to 0.45, with all 95% CI crossing zero. For models 2 and 3, panel B shows that intersectional estimates had a narrower range which ranged from -0.09 to 0.09 and all 95% CI crossed zero. This indicates that there were no significant intersectional estimates in any stratum among the children with and without cough. However, intersectional strata for <5y from Luhya, Luo and other ethnic groups, new immigrants, and households experiencing CHE are at a higher risk of cough compared to other strata.

## Discussion

In this study, we explored within an intersectionality framework how SDoH are associated with diarrhea, fever, and cough in <5y living in slums. We found that the main SDoH associated with health conditions for <5y in slums was head of household ethnic group. Odds of having each of the three-health condition were significantly higher among children from Luhya, Luo and minority ethnic groups compared with Kamba community. No significant differences in the odds were found across each of the three health conditions between Kikuyu and Kamba. Similar results were found when looking at vaccine coverage and immunisation in Nairobi slums which found that Kikuyu and Kamba have better outcomes [77–80]. In the context of our analysis, it seems that Kamba being nearest to the city have appropriated the advantages of rural-urban linkages and ownership of land in their rural homes to supplement precarious urban incomes and livelihoods compared to the Luo and the Luhya whose rural homes are quite far in Western Kenya [22, 23]. The advantage experienced by <5y from Kamba community over the Kikuyu may be related to land ownership in rural homes since a Kikuyu living in the slums typifies landlessness and abject poverty despite their history of political leadership and economic dominance in Kenya. This is further explained by previous research that showed transitions out of slum poverty were lowest among Kikuyu when compared with Kamba, Luhya, and Luo which indicate they don’t benefit from rural -urban linkages despite them bordering the city [81]. In addition, it has been shown that different cultural practices among ethnic groups influence their health seeking behavior [77–80, 82]. For example, [82] found that all other ethnic groups apart from Kamba were more likely to stop breastfeeding their infants earlier compared to Kikuyu. Early weaning may lead to undernutrition which is associated with increased burden of disease such as diarrhea [83–85]. These may also explain why children aged <2 years have higher odds of getting diarrhea compared to those aged 2 to 5 years.

Children <5y from households who have stayed in the sampled slum for more than 2 years have significantly lower odds of cough compared to those how have lived in slums for less than 2 years. The poor living conditions in slums lead to higher exposure of various risks making them less susceptible to diseases as a result of biological adaptations over time [86]. The result is consistent with the social disruption associated with migration generally more evident among new migrants especially with distressed migrants like refugees and internally displaced persons and economic migrants with slum destinations as they struggle to navigate and adapt to economic and environmental challenges of their new destination.

In addition, people who have stayed in slums over a long duration may have benefited from community training on how to improve their children’s health through various health education and interventions programs offered in the slums [87, 88]. The significant associations between a household experiencing CHE with cough and fever indicate the extra financial burden faced by these households. This is consistent with findings by [89] that showed that CHE cases were associated with fever and cough across 39 countries in LMICs. Notably, high CHE experienced by households in poverty may reduce the amount of disposable income they can spend on other subsistence costs which renders them ill-equipped to cater for nutritious food and healthcare making members of such households more susceptible to ailments [67]. Moreover, households experiencing CHE are highly likely to get into more extreme poverty due to loss of income caused by absence of taking care of the ill child which exacerbates inequalities in their health status [90]. This may explain why <5y from households with no health insurance have higher and significant odds of getting diarrhea compared to those from covered households. This indicates the need to implement social health insurance programs for slum dwellers to reduce the burden of health care costs. Notably, <5y from educated households tend to have significantly higher odds of getting diarrhea compared to those from households with no education. This is consistent with previous research by [81] and [91] that found diminishing role of education in improving economic status of households in the slums due to limited availability of formal sector jobs and high unemployment rates.

The intersectional MAIHDA approach shows evidence of intersectional disparities based on SDoHs for <5y, especially for diarrhea, providing precise information for identifying population groups for targeted intervention. For example, children aged 1 year and younger from Luhya and Luo ethnicities, new immigrants, households with no health insurance and educated were found to be at a higher risk of diarrhea compared to other groups. This was further supported by VPC and AUC-ROC measures which indicated that <5y from these intersectional stratifications were more vulnerable to diarrhea compared to others despite all being subjected to the same poor living conditions in slums. The higher values of DA obtained for <5y with diarrhea implies that vulnerable groups identified can be targeted for interventions. However, considering that intersectional effects used to identify these vulnerable groups were not significant, interventions are also supposed to be extended to other groups despite them not being more vulnerable.

The results for fever and cough are not generalisable to the slum population because of the low values of DA (i.e., as indicated by their VPC and AUC-ROC). This may have been caused by the poor definition of social strata where the variables used in creating them are not better suited in defining social positions and identities that are associated with fever and cough inequalities. Moreover, the larger number of social strata for both cough and fever resulted in many strata having few observations which may have led to unstable fixed effect estimates and therefore low DA [58]. The smaller intersectional differences for fever and cough suggest that pathways for vulnerability might be shared by SHoD considered in the models. For example, <5y from Luhya and Luo s may be experiencing worse health conditions because they are poorer and not benefiting from rural-urban linkages compared to Kamba [22, 23]. This implies that the problem may not be about head of household ethnic group, but the limited survival strategies available to this ethnic groups. Therefore, the focus should be on ways of improving wealth and living conditions of Luhya and Luo communities such as providing employment opportunities as this could lessen the ethnic inequalities observed. It is crucial to note the advantages of rural-urban linkages for slum dwellers may have lessened over time considering the financial inclusion brought about by widespread use of mobile money transfer [28]. Despite data used being more than a decade old, these findings present patterns in the intersections of various social positions and identities and contributes to understanding of health inequalities facing <5y living in slums. To our knowledge this has not being studied before and highlights specific strata which experience inequalities and help understand how various system of oppression and privilege contribute to <5y health outcomes. Moreover, available evidence over the years has shown that most health and socio -economic indicators have not significantly changed in Nairobi slums implying that the data used may be of relevance today [92].

Our findings have two implications. First, we provide new insights that <5yr from Luo and Luhya ethnic groups, recent migrants (less than 2 years) and households experiencing CHE are more likely to face worse health outcomes. Therefore, Nairobi County government which is mandated with provision of health services need to develop strategies aimed at identifying these groups for targeted proportionate universalism based on level of need [93]. This may involve engaging with community health volunteers (CHVs), community members and other stakeholders to develop health policies for <5yr across these groups and another which focuses on the whole slum community. Second, our analysis shows that application of quantitative intersectionality can help identify social positions and identities that are at higher risk of health inequalities. This is especially useful in settings such as slums where resources are already constrained which makes universal targeting difficult. Notably, application of quantitative intersectionality in such settings can be enhanced by engaging the community to identify the relevant determinants that drive health inequalities which can then be used to define social positions and identities.

Limitations to this study include firstly the passage of time since conduct of NCSS 2012: the data are 11 years old and may need to be interpreted cautiously due to the dynamic nature of slums. However, these findings remain of value since NCSS 2012 was representative of the population considered and is the most recently conducted slum survey available from Nairobi. Secondly, we conducted complete case analyses for each of health conditions, and this could have introduced selection bias if missing data were not completely at random [94]. However, it is also important to note that imputing many missing values in outcomes can raise questions as to acceptability and accuracy of the health conditions [95]. Thirdly, variables included in the analyses were selected based on univariate analysis, with variables that showed statistical significance (*p* < 0.05). One drawback of univariate analysis for variable selection is that it ignores the fact that individual variables that are weakly associated with the outcome of interest can contribute significantly to the model when they are combined with other variables [69, 96]. This problem can be solved setting a higher significance level to allow inclusion of more important variables relevant to the outcome [69]. However, we could not consider higher significance levels in our analyses because of the need to overcome the issue of the large number of strata which could have resulted to unstable estimates because the sample size of NCSS 2012 data was moderate. Importantly, future research should consider using social strata based on theorised literature and community views about determinants that influence health conditions among <5 y in slums rather than relying on model building process [97]. This will greatly enhance better understanding of inequalities patterning for effective targeted interventions.

Fourth, MAIHDA approach is only explorative because intersectional matrices are based on socio determinants factors that do not necessarily have an established theory and hypotheses for each of the intersectional strata [55, 98]. Despite MAIHDA limitation, we were able to provide inductive information on how socio determinants factors contribute to health inequalities for children <5y living in slums through intersectionality lens based on quantitative analyses. In turn, this provides an understanding of how multiple SDOH interact to create dynamics of vulnerability that lead to health inequalities amongst marginalised urban dwellers. There is also potential for bias due to very high proportion of “don’t know and not applicable” categorical levels for some variables such as education and length of stay. However, sensitivity analysis excluding “don’t know and not applicable” individuals for the health outcome diarrhea indicated no changes in the overall pattern of intersectional estimates (S3 Table and S1 Fig). There is potential bias in the estimates caused by undertaking complete case analysis if the data were not missing completely at random because about 9% of records were dropped due to missing health outcomes and another 8% due to missing explanatory variables. Finally, the study results may be biased due to low power because the sample size was estimated to measure prevalence of <5yrs health outcomes and not associations [10].

In conclusion, despite children in Nairobi slums experiencing worse conditions on the whole compared to their counterparts in other urban and rural areas; their vulnerabilities within these slums are unevenly distributed. The findings from this study clearly show that pathways of SDoH that affect <5y health conditions in slums are interlocking (i.e., multiplicative in nature and shared). This suggests that governments and other actors need to prioritise investing in programs that target specific social strata that are at a higher risk of experiencing worse health outcomes. This implies that improving health for <5y requires multiple approaches based on specific circumstances of each vulnerable group if health inequalities were to be reduced. Future studies should also consider using MAIHDA approach to explore the effect of intersecting dimensions of SDoH as this will help address health inequalities.

## Supporting information

S1 Text Description of multilevel analysis of individual heterogeneity and discriminatory accuracy (MAIHDA) model building. (DOCX)

S1 Fig Estimated intersectional effects estimates and their corresponding 95% credible intervals (CI) for each stratum for diarrhea ranked from lowest to highest: Model 1 (panel A) and model 2 (panel B)–sensitivity analysis when “Don’t know and not applicable” in education variable are included in model. (EPS)

S1 Table Distribution of socio determinants characteristics for diarrhea in Nairobi Cross-sectional survey 2012. (DOCX)

S2 Table Univariate analyses for diarrhea in Nairobi Cross-sectional survey 2012. (DOCX)

S3 Table Fixed effects, strata variance, area under the curve, variance partition coefficient and proportional change of variance for diarrhea (model 1 and 2) in Nairobi Cross-sectional Survey 2012–sensitivity analysis when “Don’t know and not applicable” in education variable are included in model. (DOCX)

S4 Table Distribution of socio determinants characteristics for fever in Nairobi Cross-sectional survey 2012. (DOCX)

S5 Table Univariate analyses results for fever in Nairobi Cross-sectional survey 2012. (DOCX)

S6 Table Distribution of socio determinants characteristics for cough in in Nairobi Cross-sectional survey 2012. (DOCX)

S7 Table Univariate analyses for cough in Nairobi Cross-sectional survey 2012. (DOCX)
