# Supplementary material for: Health inequalities at the intersection of multiple social determinants among under five children residing Nairobi urban slums: An application of multilevel analysis of individual heterogeneity and discriminatory accuracy (MAIHDA)
Source: PLOS Glob Public Health. 2024 Feb 29;4(2):e0002931. doi: 10.1371/journal.pgph.0002931 (PMC10903897; doi:10.1371/journal.pgph.0002931)
Supplement: S1 Text — (DOCX) [file pgph.0002931.s001.docx]

Supporting information: Health inequalities at the intersection of multiple social determinants among under five children residing Nairobi urban slums: an application of multilevel analysis of individual heterogeneity and discriminatory accuracy (MAIHDA).

**Statistical details**

Let$y_{ij}$ denote a binary health outcome (i.e., whether has an outcome or not) for child $i\left( i=1,\ldots,n \right)$ in intersectional strata $j \left( j=1,\ldots,N \right)$ where:

$y_{ij}=\left\{ \begin{aligned} 0 absence of health outcome \\ 1 Presence of health outcome \end{aligned} \right.$ Eq. (1)

$y_{ij}$ is assumed to follow a Bernoulli distribution, with probabilities $\pi_{ij}=Pr\left( y_{ij}=0 \right)$ the probability of child $i$ from intersectional stratus$j$having no health outcome and $1-\pi_{ij}=Pr\left( y_{i}=1 \right)$the probability of child $i$ from stratus$j$having a health outcome. Let $X_{ij}^{'}$ be a vector of social determinants of health (SDOH) used as explanatory variables. The multilevel logistic for model 1 with no main effects takes the form:

$logit\left( \pi_{ij} \right)=log\left( \frac{\pi_{ij}}{1-\pi_{ij}} \right)=\beta_{0}+\mu_{0j}$ Eq. (2)

where $\beta_{0}$ is the intercept and $\mu_{0j}\sim N\left( 0,\sigma_{\mu}^{2} \right)$ represents the random intercept for the intersectional stratum level residual which is normally distributed with mean $0$ and variance $\sigma_{\mu}^{2}$. Model 1 include explanatory variables, so the intersectional stratum random effect captures both the main effects of SDOH used to define intersectional strata and their interactions. Assuming no omitted variable bias, the intersectional strata level residual $\mu_{0j}$ captures the unique intersectional effects for each intersectional strata (i.e., intersectional -specific differences in health condition) while accounting for sample size differences for each social group.

Eq. 2 can be extended into model 2 by including main effects (i.e., SDOH used in construction intersectional strata) as explanatory variables and takes the form:

$logit\left( \pi_{ij} \right)=log\left( \frac{\pi_{ij}}{1-\pi_{ij}} \right)=\beta_{0}+X_{ij}^{'}\beta+\mu_{0j}$ Eq. (3)

where $\beta_{0}$is the intercept, $X_{ij}^{'}$is a vector of vector of SDOH used in creating intersectional strata with coefficient vector $\beta$ , and $\mu_{0j}\sim N\left( 0,\sigma_{\mu}^{2} \right)$ is a random intercept assumed to follow a normal distribution with mean $0$ and variance $\sigma_{\mu}^{2}.$

We used variance partitioning coefficient (VPC) to estimate discriminatory accuracy of intersectional strata n models 1 and 2 [1,2]. VPC indicates the share of the total individual variance in the in the probability of having a health outcome that is accounted for at the intersectional strata level [2]. VPCs were calculated for both model 1 and 2 using Equation (4):

$VPC=\left( \frac{\sigma_{\mu}^{2}}{\sigma_{\mu}^{2}+ 3.29} \right)\times100\%$ Eq. (4)

Where $\sigma_{\mu}^{2}$ denotes the between stratum variance in the propensity for having a health outcome, while 3.29 indicates the within stratum between individual stratum variance constrained equal to the variance of the standard logistic distribution [1]. VPC will be presented as the percentage share of individual variance which lies between strata. In model 2, assuming no relevant variables were omitted when constructing strata, VPC inform on the existence of intersectional multiplicative interaction effects [1,4,5].

The proportion of variance explained by the adding main effects is estimated by calculating the proportional change in variance (PCV) of intersectional strata between null model and model including fixed effects [1]

$PCV=\left( \frac{\sigma_{\mu(1)}^{2}-\sigma_{\mu(2)}^{2}}{\sigma_{\mu(1)}^{2}} \right)\times100\%$ Eq. (5)

where $\sigma_{\mu(1)}^{2}$ and $\sigma_{\mu(2)}^{2}$represents the intersectional strata variances in the null model and the model containing main effects respectively. The PCV represents the proportion of the total between-stratum variance of intersectional strata of the null model that is explained after including main effects. In the absence of any stratum specific interactions, the main effects used to construct the intersectional strata would completely explain the between stratum variance and all stratum random effects would be equal to zero. This implies that, the lower the PCV, the higher the amount explained variance which can be due to interaction effects or to omitted variable bias [1,4,5]. For model 3, we just added the explanatory variables which were not included in model 2.

## References

## 1. Merlo J, Yang M, Chaix B, Lynch J, Råstam L. A brief conceptual tutorial on multilevel analysis in social epidemiology: investigating contextual phenomena in different groups of people. Journal of Epidemiology & Community Health. 2005;59(9):729-36.

## 2. Goldstein H, Browne W, Rasbash J. Partitioning variation in multilevel models. Understanding statistics: statistical issues in psychology, education, and the social sciences. 2002;1(4):223-31.

## 3. Goldstein H. Multilevel statistical models: John Wiley & Sons; 2011.

## 4. Evans CR, Leckie G, Merlo J. Multilevel versus single-level regression for the analysis of multilevel information: The case of quantitative intersectional analysis. Soc Sci Med. 2020;245:112499.

## 5. Persmark A, Wemrell M, Zettermark S, Leckie G, Subramanian S, Merlo J. Precision public health: mapping socioeconomic disparities in opioid dispensations at Swedish pharmacies by multilevel analysis of individual heterogeneity and discriminatory accuracy (MAIHDA). PloS one. 2019;14(8):e0220322.
