# Supplementary material for: Health inequalities at the intersection of multiple social determinants among under five children residing Nairobi urban slums: An application of multilevel analysis of individual heterogeneity and discriminatory accuracy (MAIHDA)
Source: PLOS Glob Public Health. 2024 Feb 29;4(2):e0002931. doi: 10.1371/journal.pgph.0002931 (PMC10903897; doi:10.1371/journal.pgph.0002931)
Supplement: S1 Table — (DOCX) [file pgph.0002931.s003.docx]

| Variable | Categories | Diarrhea | |  |
| --- | --- | --- | --- | --- |
|  |  | Yes | No |  |
| **Children demographic characteristics** | | | | |
| Age | 1 year and less (infants) | 124 (21.3%) | 458 (78.7%) | 582 (33.5%) |
|  | 2 -5 years | 180 (15.6%) | 976 (84.4%) | 1,156 (66.5%) |
|  |  |  |  |  |
| Sex | Male | 161 (19.0%) | 687 (81.0%) | 848 (48.8%) |
|  | Female | 143 (16.1%) | 747 (83.9%) | 890 (51.2%) |
|  | | | | |
| **Women characteristics** | | | | |
| Age | 18 years and under | 25 (23.1%) | 83 (76.9%) | 108 (6.2%) |
|  | 19 years and above | 279 (17.1%) | 1,351 (82.9%) | 1,630 (93.8%) |
| Education | Primary | 164 (18.5%) | 722 (81.5%) | 886 (51.0%) |
|  | Post primary | 136 (16.5%) | 687 (83.5%) | 823 (47.4%) |
|  | None | 4 (13.8%) | 25 (86.2%) | 28 (1.7%) |
|  | | | | |
| **Head of household demographic characteristics** | | | | |
| Gender | Female | 43 (19.1%) | 182 (80.9%) | 225 (12.9%) |
|  | Male | 261 (17.3%) | 1,252 (82.7%) | 1,513 (87.1%) |
|  |  |  |  |  |
| Ethnicity | Kamba | 39 (13.3%) | 255 (86.7%) | 294 (16.9%) |
|  | Kikuyu | 46(13.9%) | 284 (86.1%) | 330 (19.0%) |
|  | Luhya | 106 (21.8%) | 380 (78.2%) | 486 (28.0%) |
|  | Luo | 72 (20.5%) | 280 (79.5%) | 352 (20.3%) |
|  | Other | 41 (14.9%) | 235 (85.1%) | 276 (15.9%) |
|  |  |  |  |  |
| Age | 17 – 24years | 25 (18.4%) | 111 (81.6%) | 136 (7.8%) |
|  | 25 -34 years | 186 (18.7%) | 807 (81.3%) | 993 (57.1%) |
|  | 35 years above | 93 (15.3%) | 516 (84.7%) | 609 (35.0%) |
|  | | | | |
| education | None | 13 (10.4%) | 112 (89.6%) | 125 (7.2%) |
|  | educated | 191 (18.1%) | 864 (81.9%) | 1,055 (60.7%) |
|  | Don’t know and not applicable | 100 (17.9%) | 458 (82.1%) | 558 (32.1%) |
|  | | | | |
| **Social Structure** | | | | |
| Wealth index | Rich | 120 (14.0%) | 740 (86.0%) | 860 (49.5%) |
|  | Middle | 86 (22.1%) | 303 (77.9%) | 389 (22.4%) |
|  | Poor | 98 (20.0%) | 391 (80.0%) | 489 (28.1%) |
|  |  |  |  |  |
| Length of stay | New migrants | 36 (25.0%) | 108 (75.0%) | 144 (8.3%) |
|  | Old migrants | 123 (18.0%) | 559 (82.0%) | 682 (39.2%) |
|  | Not applicable | 145 (15.9%) | 767 (84.1%) | 912 (52.5%) |
|  |  |  |  |  |
| Household religion | Catholic | 63 (14.8%) | 363 (85.2%) | 426 (24.5%) |
|  | Protestant | 220 (19.3%) | 921 (80.7%) | 1,141 (65.7%) |
|  | Other | 21 (12.3%) | 150 (87.7%) | 171 (9.8%) |
|  |  |  |  |  |
| Disability in household | Yes | 6 (25.0%) | 18 (75.0%) | 20 (1.4%) |
|  | No | 282 (17.8%) | 1,299 (82.2%) | 1,581 (91.0%) |
|  | Missing/Not applicable | 16 (12.0%) | 117 (88.0%) | 133 (7.7%) |
|  |  |  |  |  |
| Tenure | No rent paid | 9 (8.7%) | 95 (91.3%) | 104 (6.0%) |
|  | Pays rent | 295 (18.1%) | 1,339 (81.9%) | 1,634 (94.0%) |
|  |  |  |  |  |
| Food availability | enough | 51 (13.5%) | 326 (86.5%) | 377 (21.7%) |
|  | not enough | 253 (18.6%) | 1,108 (81.4%) | 1,361 (78.3%) |
|  |  |  |  |  |
| Income generating activity | Employed | 94 (19.7%) | 383 (80.3%) | 477 (27.4%) |
|  | Own business | 22 (14.2%) | 133 (85.8%) | 155 (8.9%) |
|  | Not applicable | 188 (17.0%) | 918 (83.0%) | 1,106 (63.6%) |
|  |  |  |  |  |
| Health Insurance | Yes | 69 (13.7%) | 436 (86.3%) | 505 (29.1%) |
|  | No | 235 (19.1%) | 998 (80.9%) | 1,233 (70.9%) |
|  |  |  |  |  |
| health catastrophic costs | No | 276 (17.2%) | 1,324 (82.8%) | 1,600 (92.1%) |
|  | Yes | 28 (20.3%) | 110 (79.7%) | 138 (7.9%) |
| Total |  | 304 (17.5%) | 1,434 (82.5%) | 1,738 (100.0%) |
