# Supplementary material for: Health inequalities at the intersection of multiple social determinants among under five children residing Nairobi urban slums: An application of multilevel analysis of individual heterogeneity and discriminatory accuracy (MAIHDA)
Source: PLOS Glob Public Health. 2024 Feb 29;4(2):e0002931. doi: 10.1371/journal.pgph.0002931 (PMC10903897; doi:10.1371/journal.pgph.0002931)
Supplement: S2 Table — (DOCX) [file pgph.0002931.s004.docx]

|  |  | Coefficient | Standard Error | P-value |
| --- | --- | --- | --- | --- |
| Intercept | Category (reference) |  |  |  |
| Child age | 1 year and less (ref) |  |  |  |
|  | 2 -5 years | -0.39 | 0.14 | 0.01*** |
|  | | | | |
| Child Sex | Female (ref) |  |  |  |
|  | Male | 0.20 | 0.13 | 0.25 |
|  | | | | |
| Head of household sex | Female (ref) |  |  |  |
|  | Male | -0.13 | 0.18 | 0.49 |
|  | | | | |
| Head of household age | 17 – 24 (ref) |  |  |  |
|  | 25 -34 | 0.03 | 0.25 | 0.92 |
|  | 35 and above | -0.22 | 0.24 | 0.37 |
|  | | | | |
| Head of household ethnicity | Kamba (ref) |  |  |  |
|  | Kikuyu | 0.06 | 0.24 | 0.81 |
|  | Luhya | 0.60 | 0.20 | 0.01** |
|  | Luo | 0.52 | 0.22 | 0.01** |
|  | Other | 0.13 | 0.24 | 0.56 |
|  | | | | |
| Wealth index | Rich (ref) |  |  |  |
|  | Middle | 0.56 | 0.16 | 0.01*** |
|  | Poor | 0.44 | 0.15 | 0.01*** |
|  | | | | |
| Length of stay | New migrants (ref) |  |  |  |
|  | Missing | -0.57 | 0.21 | 0.01** |
|  | Old migrants | -0.42 | 0.22 | 0.06 |
|  |  |  |  |  |
| Health insurance | No (ref) |  |  |  |
|  | Yes | -0.40 | 0.15 | 0.01*** |
|  |  |  |  |  |
| Catastrophic health costs | No (ref) |  |  |  |
|  | Yes | 0.20 | 0.22 | 0.37 |
|  |  |  |  |  |
| Food security8\| | Enough (ref) |  |  |  |
|  | Not enough | 0.37 | 0.17 | 0.02* |
|  |  |  |  |  |
| Income generating activity | Employed (ref) |  |  |  |
|  | Missing/Not applicable | -0.18 | 0.14 | 0.20 |
|  | Own business | -0.39 | 0.26 | 0.13 |
|  |  |  |  |  |
| Highest Education | None (ref) |  |  |  |
|  | educated | 0.63 | 0.32 | 0.04* |
|  | Don’t know/not applicable | 0.64 | 0.30 | 0.03* |
|  |  |  |  |  |
| Religion | Catholic (ref) |  |  |  |
|  | Protestant | 0.32 | 0.16 | 0.04* |
|  | Other | -0.22 | 0.27 | 0.43 |
|  |  |  |  |  |
| Disability | Missing/not applicable (ref) |  |  |  |
|  | No | 0.46 | 0.28 | 0.09 |
|  | Yes | 0.89 | 0.54 | 0.10 |
|  |  |  |  |  |
| Women age | 18 years and below (ref) |  |  |  |
|  | 19 years and over | -0.38 | 0.24 | 0.11 |
|  |  |  |  |  |
| Women education | Primary (ref) |  |  |  |
|  | Post primary | -0.14 | 0.13 | 0.28 |
|  | None | -0.35 | 0.55 | 0.52 |
| Tenure | No rent paid (ref) |  |  |  |
|  | Pays rent | 0.84 | 0.35 | 0.02** |
