# Supplementary material for: Health inequalities at the intersection of multiple social determinants among under five children residing Nairobi urban slums: An application of multilevel analysis of individual heterogeneity and discriminatory accuracy (MAIHDA)
Source: PLOS Glob Public Health. 2024 Feb 29;4(2):e0002931. doi: 10.1371/journal.pgph.0002931 (PMC10903897; doi:10.1371/journal.pgph.0002931)
Supplement: S3 Table — (DOCX) [file pgph.0002931.s005.docx]

|  |  | Model 1 |  | Model 2 |  |
| --- | --- | --- | --- | --- | --- |
|  | Category (reference) | Odd Ratio | 95% CI | Odd Ratio | 95% CI |
| Intercept |  | 0.18** | (0.14, 0.22) | 0.03 | (0.01, 0.11) |
| Child age | 1 year and less (ref) |  |  |  |  |
|  | 2 -5 years |  |  | 0.78 | (0.54, 1.15) |
|  |  |  |  |  |  |
| Ethnic group | Kamba (ref) |  |  |  |  |
|  | Kikuyu |  |  | 0.88 | (0.44, 1.15) |
|  | Luhya |  |  | 1.94** | (1.10, 3.56) |
|  | Luo |  |  | 1.35 | (0.74, 2.52) |
|  | Other |  |  | 1.03 | (0.53, 199) |
|  |  |  |  |  |  |
| Wealth index | Rich (ref) |  |  |  |  |
|  | Middle |  |  | 1.64** | (1.04, 2.60) |
|  | Poor |  |  | 1.09 | (0.96, 1.97) |
|  |  |  |  |  |  |
| Length of stay | New migrants (ref) |  |  |  |  |
|  | Missing/not applicable |  |  | 0.65 | (0.40, 1.05) |
|  | Old migrants |  |  | 0.73** | (0.45, 1.21) |
|  |  |  |  |  |  |
| Health insurance | No (ref) |  |  |  |  |
|  | Yes |  |  | 0.70** | (0.49, 0.96) |
|  |  |  |  |  |  |
| Religion | Catholic (ref) |  |  |  |  |
|  | Protestants |  |  | 1.37 | (0.90, 2.13) |
|  | Other/not applicable |  |  | 0.90 | (0.36, 2.13) |
|  |  |  |  |  |  |
| Food security | Enough (ref) |  |  |  |  |
|  | Not enough |  |  | 1.56 | (0.97, 2.56) |
|  |  |  |  |  |  |
| Tenure | No rent (ref) |  |  |  |  |
|  | Rented |  |  | 2.07 | (0.79, 6.50) |
|  |  |  |  |  |  |
| Education | none (ref) |  |  |  |  |
|  | educated |  |  | 2.44** | (1.27, 5.04) |
|  |  |  |  |  |  |
| Strata variance |  | 0.40 |  | 0.32 |  |
| Strata N |  | 491 |  | 491 |  |
| Individual |  | 1,180 |  | 1,738 |  |
|  |  |  |  |  |  |
| AUC -ROC |  | 87.17% |  | 76.73% |  |
| VPC |  | 11.26% |  | 9.57% |  |
| PCV |  |  |  | 16.59% |  |
