# Supplementary material for: Health inequalities at the intersection of multiple social determinants among under five children residing Nairobi urban slums: An application of multilevel analysis of individual heterogeneity and discriminatory accuracy (MAIHDA)
Source: PLOS Glob Public Health. 2024 Feb 29;4(2):e0002931. doi: 10.1371/journal.pgph.0002931 (PMC10903897; doi:10.1371/journal.pgph.0002931)
Supplement: S4 Table — (DOCX) [file pgph.0002931.s006.docx]

| Variable | Categories | Fever | |  |
| --- | --- | --- | --- | --- |
|  |  | Yes | No |  |
| **Children demographic characteristics** | | | | |
| Age | 1 year and less (infants) | 110 (18.9%) | 473 (81.1%) | 583 (33.7%) |
|  | 2 -5 years | 186 (16.2%) | 962 (83.8%) | 1,148 (66.3%) |
|  |  |  |  |  |
| Sex | Male | 130 (15.3%) | 719 (84.7%) | 849 (49.0%) |
|  | Female | 166 (18.8%) | 716 (81.2%) | 882 (51.0%) |
|  | | | | |
| **Women characteristics** | | | | |
| Age | 18 years and under | 27 (24.3%) | 84 (75.7%) | 111 (6.4%) |
|  | 19 years and above | 269 (16.6%) | 1,351 (83.4%) | 1,620 (9.4%) |
| Education | Primary | 157 (17.8%) | 723 (82.3%) | 880 (50.8%) |
|  | Post primary | 139 (16.9%) | 684 (83.1%) | 823 (47.5%) |
|  | None | 0 (0.0%) | 28 (100.0%) | 28 (1.6%) |
|  | | | | |
| **Head of household demographic characteristics** | | | | |
| Gender | Female | 33 (15.1%) | 185 (84.9%) | 218 (12.6%) |
|  | Male | 263 (17.4%) | 1,250 (82.6%) | 1,513 (87.4%) |
|  |  |  |  |  |
| Ethnicity | Kamba | 28 (9.6%) | 265 (90.4%) | 293 (16.9%) |
|  | Kikuyu | 42 (12.8%) | 285 (87.2%) | 327 (18.9%) |
|  | Luhya | 110 (22.7%) | 375 (77.3%) | 485 (28.0%) |
|  | Luo | 62 (17.7%) | 288 (82.3%) | 350 (20.2%) |
|  | Other | 54 (19.6%) | 222 (80.4%) | 276 (15.9%) |
|  |  |  |  |  |
| Age | 17 – 24years | 27 (20.1%) | 107 (79.9%) | 134 (7.7%) |
|  | 25 -34 years | 180 (18.2%) | 809 (81.8%) | 989 (57.1%) |
|  | 35 years above | 89 (14.6%) | 519 (85.4%) | 608 (35.1%) |
|  | | | | |
| education | None | 23 (18.9%) | 99 (81.1%) | 122 (7.0%) |
|  | Educated | 181 (17.4%) | 862 (82.6%) | 1,043 (60.3%) |
|  | Don’t know and not applicable | 92 (16.3%) | 474 (83.7%) | 566 (32.7%) |
|  | | | | |
| **Social Structure** | | | | |
| Wealth index | Rich | 123 (14.5%) | 728 (85.5%) | 851 (49.2%) |
|  | Middle | 80 (20.5%) | 311 (79.5%) | 391 (22.6%) |
|  | Poor | 93 (19.0%) | 396 (81.0%) | 489 (28.2%) |
|  |  |  |  |  |
| Length of stay | New migrants | 34 (23.4%) | 111 (76.6%) | 145 (8.4%) |
|  | Old migrants | 123 (18.3%) | 549 (81.7%) | 672 (38.8%) |
|  | Not applicable | 139 (15.2%) | 775 (84.8%) | 914 (52.8%) |
|  |  |  |  |  |
| Household religion | Catholic | 74 (17.5%) | 348 (82.5%) | 422 (24.4%) |
|  | Protestant | 197 (17.3%) | 943 (82.7%) | 1,140 (65.9%) |
|  | Other | 25 (14.8%) | 144 (85.2%) | 169 (9.8%) |
|  |  |  |  |  |
| Disability in household | Yes | 5 (20.8%) | 19 (79.2%) | 24 (1.4%) |
|  | No | 270 (17.2%) | 1,302 (82.8%) | 1,572 (90.8%) |
|  | Missing/Not applicable | 21 (15.6%) | 114 (84.4%) | 135 (7.8%) |
|  |  |  |  |  |
| Tenure | No rent paid | 19 (18.1%) | 86 (81.9%) | 105 (6.1%) |
|  | Pays rent | 277 (17.0%) | 1,349 (83.0%) | 1,626 (93.9%) |
|  |  |  |  |  |
| Food security | enough | 49 (13.2%) | 323 (86.8%) | 372 (21.5%) |
|  | Not enough | 247 (18.2%) | 1,112 (81.8%) | 1,359 (78.5%) |
|  |  |  |  |  |
| Income generating activity | Employed | 78 (16.6%) | 393 (83.4%) | 471 (27.2%) |
|  | Own business | 25 (16.2%) | 129 (83.8%) | 154 (8.9%) |
|  | Not applicable | 193 (17.5%) | 913 (82.5%) | 1,106 (63.9%) |
|  |  |  |  |  |
| Health Insurance | Yes | 80 (15.9%) | 423 (84.1%) | 503 (29.1%) |
|  | No | 216 (17.6%) | 1,012 (82.4%) | 1,228 (70.9%) |
|  |  |  |  |  |
| health catastrophic costs | No | 260 (16.3%) | 1,335 (83.7%) | 1,595 (92.1%) |
|  | Yes | 36 (26.5%) | 100 (73.5%) | 136 (7.9%) |
| Total |  | 296 (17.1%) | 1,435 (82.9%) | 1,731 (100.0%) |
