# Supplementary material for: Health inequalities at the intersection of multiple social determinants among under five children residing Nairobi urban slums: An application of multilevel analysis of individual heterogeneity and discriminatory accuracy (MAIHDA)
Source: PLOS Glob Public Health. 2024 Feb 29;4(2):e0002931. doi: 10.1371/journal.pgph.0002931 (PMC10903897; doi:10.1371/journal.pgph.0002931)
Supplement: S6 Table — (DOCX) [file pgph.0002931.s008.docx]

| Variable | Categories | Cough | |  |
| --- | --- | --- | --- | --- |
|  |  | Yes | No |  |
| **Children demographic characteristics** | | | | |
| Age | 1 year and less (infants) | 153 (26.6%) | 423 (73.4%) | 576 (33.5%) |
|  | 2 -5 years | 277 (24.2%) | 868 (67.2%) | 1,145 (66.5%) |
|  |  |  |  |  |
| Sex | Male | 202 (24.0%) | 638 (76.0%) | 840 (48.8%) |
|  | Female | 228 (25.9%) | 653 (74.1%) | 881 (51.2%) |
|  | | | | |
| **Women characteristics** | | | | |
| Age | 18 years and under | 29 (26.9%) | 79 (73.1%) | 108 (6.3%) |
|  | 19 years and above | 401 (24.9%) | 1,212 (75.1%) | 1,613 (93.7%) |
| Education | Primary | 222(25.4%) | 651(74.6%) | 873 (50.7%) |
|  | Post primary | 205 (25.1%) | 613 (74.9%) | 818 (47.5%) |
|  | None | 3 (10.0%) | 27 (90.0%) | 30 (17.0%) |
|  | | | | |
| **Head of household demographic characteristics** | | | | |
| Gender | Female | 50 (22.5%) | 172 (77.5%) | 222 (12.9%) |
|  | Male | 380 (25.4%) | 1,119 (74.5%) | 1,499 (87.1%) |
|  |  |  |  |  |
| Ethnicity | Kamba | 44(15.0%) | 250 (85.0%) | 294 (17.1%) |
|  | Kikuyu | 70 (21.4%) | 257 (78.6%) | 327 (19.0%) |
|  | Luhya | 144 (30.0%) | 336 (70.0%) | 480 (27.9%) |
|  | Luo | 96 (27.7%) | 250 (42.3%) | 346 (20.1%) |
|  | Other | 76 (17.7%) | 198 (72.3%) | 274 (15.9%) |
|  |  |  |  |  |
| Age | 17 – 24years | 35 (25.9%) | 100 (74.1%) | 135 (7.8%) |
|  | 25 -34 years | 264 (26.9%) | 719 (73.1%) | 983 (57.1%) |
|  | 35 years above | 131 (21.7%) | 472 (78.3%) | 603 (35.0%) |
|  | | | | |
| education | None | 26 (21.5%) | 95 (78.5%) | 121 (7.0%) |
|  | educated | 263 (25.1%) | 784 (74.9%) | 1,047 (60.8%) |
|  | Don’t know and not applicable | 141 (27.4%) | 412 (74.5%) | 553 (32.1%) |
|  | | | | |
| **Social Structure** | | | | |
| Wealth index | Rich | 188 (22.2%) | 658 (77.8%) | 846 (49.2%) |
|  | Middle | 108 (27.9%) | 279 (72.1%) | 387 (22.5%) |
|  | Poor | 134 (27.5%) | 354 (72.5%) | 488 (28.4%) |
|  |  |  |  |  |
| Length of stay | New migrants | 50 (34.7%) | 94 (65.3%) | 144 (8.4%) |
|  | Old migrants | 162 (23.9%) | 515 (76.1%) | 677 (39.3%) |
|  | Missing/Not applicable | 218 (24.2%) | 682 (75.8%) | 900 (52.3%) |
|  |  |  |  |  |
| Household religion | Catholic | 101 (24.05%) | 319 (76.0%) | 420 (24.4%) |
|  | Protestant | 293 (25.8%) | 842 (74.2%) | 1,135 (66.0%) |
|  | Other | 36 (21.7%) | 130 (78.3%) | 166 (9.6%) |
|  |  |  |  |  |
| Disability | No | 390 (25.0%) | 1,173 (75.0%) | 1,563 (90.8%) |
|  | Yes | 8 (33.3%) | 16 (66.7%) | 24 (1.4%) |
|  | Missing/Not applicable | 32 (23.9%) | 102 (76.1%) | 134 (7.8%) |
|  |  |  |  |  |
| Tenure | No rent paid | 20 (19.4%) | 83 (80.6%) | 103 (60.0%) |
|  | Pays rent | 410 (25.3%) | 1,208 (74.7%) | 1,208 (74.7%) |
|  |  |  |  |  |
| Food security | enough | 87 (23.5%) | 284 (76.5%) | 371 (21.6%) |
|  | not enough | 343 (25.4%) | 1,007 (74.6%) | 1,350 (78.4%) |
|  |  |  |  |  |
| Income generating activity | Employed | 120 (25.3%) | 355 (74.7%) | 475 (27.6%) |
|  | Own business | 38 (24.4%) | 118 (75.6%) | 156 (9.1%) |
|  | Not applicable | 272 (25.0%) | 818 (75.0%) | 1,090 (60.5%) |
|  |  |  |  |  |
| Health Insurance | Yes | 130 (26.1%) | 368 (73.9%) | 498 (28.9%) |
|  | No | 300 (24.5%) | 923 (75.5%) | 1,223 (71.1%) |
|  |  |  |  |  |
| health catastrophic costs (40% threshold) | No | 380 (24.0%) | 1,201 (76.0%) | 1,581 (91.9%) |
|  | Yes | 50 (35.7%) | 90 (64.3%) | 140 (7.7%) |
| Total |  | 430 (25.0%) | 1,291 (75.0%) | 1,721 (100.0%) |
