# Supplementary material for: Health inequalities at the intersection of multiple social determinants among under five children residing Nairobi urban slums: An application of multilevel analysis of individual heterogeneity and discriminatory accuracy (MAIHDA)
Source: PLOS Glob Public Health. 2024 Feb 29;4(2):e0002931. doi: 10.1371/journal.pgph.0002931 (PMC10903897; doi:10.1371/journal.pgph.0002931)
Supplement: S7 Table — (DOCX) [file pgph.0002931.s009.docx]

|  | |  |  | Coefficient | Standard Error | P-value |
| --- | --- | --- | --- | --- | --- | --- |
| Intercept | | Category (reference) |  |  |  |  |
| Child age | | 1 year and less (ref) |  |  |  |  |
|  |  | 2 -5 years |  | -0.13 | 0.12 | 0.30 |
|  |  | | | | | |
| Child Sex | | Female (ref) |  |  |  |  |
|  |  | Male |  | -0.10 | 0.11 | 0.38 |
|  |  | | | | | |
| Head of household sex | | Female (ref) |  |  |  |  |
|  |  | Male |  | 0.16 | 0.17 | 0.36 |
|  |  | | | | | |
| Head of household age | | 17 – 24 (ref) |  |  |  |  |
|  |  | 25 -34 |  | 0.05 | 0.21 | 0.82 |
|  |  | 35 and above |  | -0.23 | 0.22 | 0.29 |
|  |  | | | | | |
| Head of household ethnicity | | Kamba (ref) |  |  |  |  |
|  |  | Kikuyu |  | 0.44 | 0.21 | 0.04* |
|  |  | Luhya |  | 0.89 | 0.19 | 0.01** |
|  |  | Luo |  | 0.78 | 0.20 | 0.01** |
|  |  | Other |  | 0.78 | 0.21 | 0.01** |
|  |  | | | | | |
| Wealth index | | Rich (ref) |  |  |  |  |
|  |  | Middle |  | 0.30 | 0.14 | 0.03* |
|  |  | Poor |  | 0.28 | 0.13 | 0.03* |
|  |  | | | | | |
| Length of stay | | New migrants (ref) |  |  |  |  |
|  |  | Missing |  | -0.51 | 0.19 | 0.01** |
|  |  | Old migrants |  | -0.53 | 0.20 | 0.07** |
|  | |  |  |  |  |  |
| Health insurance | | No (ref) |  |  |  |  |
|  |  | Yes |  | 0.08 | 0.12 | 0.49 |
|  | |  |  |  |  |  |
| Catastrophic health expenditure | | No (ref) |  |  |  |  |
|  |  | Yes |  | 0.56 | 0.18 | 0.01** |
|  | |  |  |  |  |  |
| Food security | | Enough (ref) |  |  |  |  |
|  |  | Not enough |  | 0.11 | 0.14 | 0.44 |
|  | |  |  |  |  |  |
| Income generating activity | | Employed (ref) |  |  |  |  |
|  |  | Missing/Not applicable |  | -0.02 | 0.13 | 0.90 |
|  |  | Own business |  | -0.05 | 0.21 | 0.82 |
|  | |  |  |  |  |  |
| Highest Education | | None (ref) |  |  |  |  |
|  |  | educated |  | 0.22 | 0.24 | 0.36 |
|  |  | Don’t know/not applicable |  | 0.20 | 0.23 | 0.38 |
|  | |  |  |  |  |  |
| Religion | | Catholic (ref) |  |  |  |  |
|  |  | Protestant |  | -0.13 | 0.22 | 0.54 |
|  |  | Other |  | 0.09 | 0.13 | 0.48 |
|  | |  |  |  |  |  |
| Disability | | No (ref) |  |  |  |  |
|  | | Missing/not applicable |  | -0.05 | 0.21 | 0.78 |
|  | | Yes |  | 0.40 | 0.43 | 0.35 |
|  | |  |  |  |  |  |
| Women age | | 18 years and below (ref) |  |  |  |  |
|  | | 19 – 49 years |  | -0.10 | 0.22 | 0.64 |
| Women education | | Primary (ref) |  |  |  |  |
|  | | Post primary |  | -0.02 | 0.11 | 0.86 |
|  | | None |  | -1.12 | 0.61 | 0.07 |
|  | |  |  |  |  |  |
| Tenure | | No rent paid (ref) |  |  |  |  |
|  | | Pays rent |  | 0.34 | 0.25 | 0.18 |

. ** P<.05: significant
